# Wasting the doctor's time? A video-elicitation interview study with patients in primary care

**DOI:** 10.1016/j.socscimed.2017.01.025

**Published:** 2017-03

**Authors:** Nadia Llanwarne, Jennifer Newbould, Jenni Burt, John L. Campbell, Martin Roland

**Affiliations:** a Cambridge Centre for Health Services Research, Primary Care Unit, Institute of Public Health, Forvie Site, University of Cambridge School of Clinical Medicine, Box 113 Cambridge Biomedical Campus, Cambridge, CB2 0SR, UK; b University of Exeter Medical School, St Lukes Campus, Magdalen Road, Exeter, EX1 2LU, UK

**Keywords:** United Kingdom, Patient experience, Help seeking, Primary care, Candidacy, Video elicitation interviews, Wasting time, Access

## Abstract

Reaching a decision about whether and when to visit the doctor can be a difficult process for the patient. An early visit may cause the doctor to wonder why the patient chose to consult when the disease was self-limiting and symptoms would have settled without medical input. A late visit may cause the doctor to express dismay that the patient waited so long before consulting. In the UK primary care context of constrained resources and government calls for cautious healthcare spending, there is all the more pressure on both doctor and patient to meet only when necessary. A tendency on the part of health professionals to judge patients' decisions to consult as appropriate or not is already described. What is less well explored is the patient's experience of such judgment. Drawing on data from 52 video-elicitation interviews conducted in the English primary care setting, the present paper examines how patients seek to legitimise their decision to consult, and their struggles in doing so. The concern over wasting the doctor's time is expressed repeatedly through patients' narratives. Referring to the sociological literature, the history of ‘trivia’ in defining the role of general practice is discussed, and current public discourses seeking to assist the patient in developing appropriate consulting behaviour are considered and problematised. Whilst the patient is expected to have sufficient insight to inform timely consulting behaviour, it becomes clear that any attempt on the part of doctor or patient to define legitimate help-seeking is in fact elusive. Despite this, a significant moral dimension to what is deemed appropriate consulting by doctors and patients remains. The notion of candidacy is suggested as a suitable framework and way forward for encompassing these struggles to negotiate eligibility for medical time.

## Highlights

• The study considers patients' worry about wasting doctors' time in UK primary care.
• Doctors and public campaigns refer to appropriate and inappropriate service users.
• Patients adopt a dichotomised view of rational users and ‘timewasters’.
• Beneath lies real uncertainty over what constitutes a good enough reason to consult.
• Candidacy offers a helpful framework to negotiate eligibility for medical time.

## 1 Introduction

The timing of the first consultation between the primary care doctor and the patient marks the beginning of the patient's journey through the healthcare system, and determines if and when a diagnosis occurs, and whether treatments or referrals ensue (Morgan, 2003). If patients present early in the natural course of the disease, symptoms may be vague and mild, and the recommendation offered to the patient is often to watch and wait. If the illness is thought to be self-limiting, the recommendation is patience, with advice on self-care. If symptoms are established and clinical signs elicited by the doctor, medical action might be taken in the form of medication, investigation or referral. Finally, if the symptoms have a long history or are interpreted by the doctor as suggesting underlying serious disease, a fast-track referral may be made. In the context of UK primary care, general practitioners (GPs) hold a gatekeeping role to triage and select those few patients who require further investigation and referral, amongst a majority for whom it is appropriate to offer advice, reassurance, watchful waiting or treat in primary care. This gatekeeping role is key to ensuring overall efficiency of the system and avoiding unnecessary medical interventions (Starfield et al., 2005).

Rationing is an inherent component of the British healthcare service (Mechanic, 1995), and general practitioners in particular are aware of the financial constraints within which they must operate (Jones et al., 2004). Increasing demand resulting from shifting demographics and advancing technology contributes to added pressure on the health service to control cost.

Central to this pressure for efficiency is ensuring that time is optimally used (Williams, 1998). With time experienced as a scarce commodity requiring thoughtful allocation (Horobin and McIntosh, 1983), patients with unexplained or self-limiting symptoms are at risk of being viewed by healthcare providers as drawing resources away from those patients more in need. Consultations for what were labelled ‘trivial conditions’ were already reported in 1964 as the greatest source of frustration in a UK-wide survey of GPs (Cartwright, 1967). More recent evidence suggests this frustration persists (Morris et al., 2001, Majid, 2015). Faced with this frustration, doctors may intuitively assign moral value to patients' reasons for help-seeking. Moral labelling, according to the sociologist Phil Strong, does not typically occur publicly: “A fundamental premise of normal doctor patient interaction is that, at least overtly, the patient is assumed to possess considerable moral character and competence” (Strong, 1979a). In his study of paediatric clinics, Strong describes a bureaucratic form where a semblance of moral neutrality dominates the clinic, and the patient (or in this case the parent) is idealised (Strong, 1979b). However, alongside this polite format, he observes what he calls a ‘charitable’ form in which moral judgments of parents are cast readily by doctors. Such judgments have been documented in the emergency department (Hillman, 2014, Jeffery, 1979, Roth, 1972) and in general practice (Charles-Jones et al., 2003, May et al., 2004). This moral labelling of patients by doctors takes many forms. It may relate to the patient's social deservedness (whether the patient is deemed responsible for the ailments), to the legitimacy of the patient's symptoms (whether the symptoms are deemed by the doctor to be organic or imagined) (Roth, 1972), or to a moral judgment on the appropriateness of health service use (Jeffery, 1979). It is the moral dimension of help-seeking which we focus on here. Most researchers report the phenomenon based on interviews with doctors (Charles-Jones et al., 2003, May et al., 2004), and on observations of consultations (Roth, 1972, Jeffery, 1979, Strong, 1979b).

If the prevailing moral labelling is sufficiently overt to be perceived by researchers, to what extent is it apparent to patients? How does this judgment influence patients' decisions to consult? Although it is said that, for a long while, patients were sheltered from the economic dimension of healthcare provision, pressures on resources have gradually become more explicit (Hughes and Griffiths, 1997, Russell et al., 2011). Public campaigns ask patients to refrain from using services unnecessarily (Choose well this winter, 2013). So how do patients experience this pressure to ‘choose well’? Worries about wasting the doctor's time are frequently touched on in studies examining barriers to help-seeking, in particular amongst parents consulting with children (Cabral et al., 2015, Usher-Smith et al., 2015), and amongst patients with possible symptoms of cancer (Walter et al., 2014, Low et al., 2015). Only very recently has it become a subject of study in its own right (Cromme et al., 2016).

This paper devotes itself to investigating the patient's account of negotiating service use, and in particular the voiced notion of ‘wasting the doctor's time’ in UK general practice. The subject arose from interviews conducted with patients exploring their experience of a recent primary care consultation. The ‘wasting doctors’ time’ theme lay beyond the primary aims of the original research and was not purposely explored during the interviews. However it arose sufficiently forcefully during data collection and preliminary analyses of early interviews to afford study in its own right. The purpose here is to investigate this moral component voiced in patients' accounts of help-seeking, situating it within the current social and political climate. Owing to the surface moral neutrality of medicine which Strong describes, the moral dimension of help-seeking has been broadly overlooked in biomedicine, and it remains absent from many psychological models. We suggest that the theoretical notion of candidacy can be applied in conceptualising the moral component of help-seeking. Candidacy is a staged model of healthcare access which traces the patient journey from first noticing a need to consult, to the concluding encounters between patient and health service (Dixon-Woods et al., 2006). In recognising the adjudication by health professionals to which patients are exposed, and emphasising the process of negotiating entitlement to care, candidacy acknowledges the patient's worry about timewasting, and offers a framework accessible across disciplinary boundaries. It thus provides opportunity for insight into important components of the consultation which should be of interest to social scientists and clinicians alike. Accordingly, we aim to give attention to the concerns among patients about wasting doctors' time, and understand the contributing factors to such concerns. Studying these concerns is a crucial aspect of the endeavour to overcome barriers to healthcare.

## 2 Methods

### 2.1 Sampling and recruitment

This study is part of a wider programme of research investigating the role of patient experience surveys in primary care. The data presented are derived from video-elicitation interviews which were conducted with the aim of exploring patients' experiences of a recent consultation in primary care, with a particular focus on how these experiences related to their completion of a questionnaire on doctors' communication skills.

GP practices were sampled purposively to reflect a spread of practice characteristics, including size and geographical location, and a mix of ethnicity and deprivation levels. Sampling also took account of practice-level scoring on the doctor-patient communication items of the national GP Patient Survey. Patient experience scores in the national survey are typically high. To optimise access to a wider range of communication scores in line with the primary aims of the research programme (Roland et al., In Press) we intentionally only included practices scoring in the bottom 25% nationally. Following consent from doctors and patients, consultations were video-recorded. Immediately after the consultation, patients filled out a short survey (box 1) on their experience of the doctor's communication skills. Patients who expressed interest in taking part in an interview were subsequently contacted by a researcher by telephone or email. Patients were selected for invitation to interview according to a maximum variation sampling approach, to reflect a mix of patient characteristics and patient experience scores reported following the consultation.

### 2.2 Interview procedure

Interviews took place between August 2012 and July 2014, within four weeks of the consultation with the GP. 44 interviews were conducted in the participant's home, six at the GP surgery, one on university premises, and one at the participant's place of work. Interviews were semi-structured and focussed on the patient's recently recorded consultation with their GP. The interview was conducted using video-elicitation methods (Henry and Fetters, 2012). The technique involves playing the video of the patient's consultation with their GP during the interview (box 2). The video becomes a central feature in guiding the interview, and points of discussion arise whilst watching the interactions between doctor and patient. Participants are encouraged to pause the recording when the viewing triggers a thought or comment. The aim is to facilitate recall of the consultation and reflection on events, through re-living of the consultation (Henry and Fetters, 2012). In qualitative research, filming of everyday life has a long history in ethnographic research, and is increasingly being used in conversation analysis of contemporary social interactions (Heath and Luff, 2008). Likewise, video-recording consultations has become a recognised research method for studying the doctor-patient relationship (Arborelius and Timpka, 1990, Arborelius et al., 1992). In studying the complexity of consultations between doctor and patient, video-elicitation interviews offer the combined benefits of both constituent methods: the interview brings depth and flexibility; the video-recording brings context which facilitates dissection of specific components of the consultation. Together, they provide a powerful means of generating rich narratives of patient experience (Cromarty, 1996, Coleman and Murphy, 1999). In the context of this paper particularly, the stimulus afforded by the direct viewing of the event allowed for exploration of unanticipated themes. In addition, the focus on a single consultation provided by the video-observation led to discussions about help-seeking.

Following written consent from the participants, interviews were audio-recorded. Recordings were transcribed verbatim. Transcripts were anonymised and checked against the audio recording for accuracy of transcription.

The research was approved by the regional ethics committee, the NRES Committee East of England, in October 2011 (ref: 11/EE/0353).

### 2.3 Analysis

Analysis was iterative and inductive in line with qualitative principles (Pope et al., 2000), but broadly took place in two phases.

The first phase involved detailed analysis by the first author (NL) of a sample of 12 interviews. During the process of interviewing and parallel familiarisation with the assembled texts, video, and audio-recordings, the first author identified ‘wasting the doctor's time’ as a prominent feature in patients' accounts. This subject arose recurrently during interviews despite its absence in the interview schedule, thus qualifying as an *emergent* theme, in contrast to what Ziebland et al. have termed *anticipated* themes (Ziebland and McPherson, 2006). The structure of the interview followed the storyline of the recorded consultation watched on the screen, and the order of the statements on the patient questionnaire (box 1). The theme of wasting the doctor's time typically emerged when discussing the reason for consulting, or when discussing questionnaire items relating to ‘giving you enough time’, ‘being treated with care and concern’ and ‘taking your problems seriously’. The reflective nature of the interview promoted wider discussion around this subject. NL developed specific codes to draw on this theme, and proceeded with categorisation and analysis of the selected 12 interviews. Indexing of the material was achieved through NVivo10 software. All the transcripts were read and re-read by the first and second authors (NL, JN), and successive stages of analysis were discussed over several consensus meetings between the first, second and third authors (NL, JN, JB). The second phase of the analysis involved expanding the dataset to study all 52 interviews conducted by four different researchers (NL, JN, AD and ET - see acknowledgement). Preliminary hypotheses arising from the first stage of in-depth analysis were tested against the remaining interviews. Following on from this process, overarching ideas were reiterated, and theories were refined to encompass the added subtleties derived from a larger dataset. Deviant cases (Lewis and Ritchie, 2003) were identified and discussed during consensus meetings. Pseudonyms were assigned to all participants. Public campaigns running in parallel were drawn on to contextualise the findings (Choose well this winter, 2013, When to see your GP, 2014). The overall relationship between the communication questionnaire scores and the video-elicitation interviews is the subject of the primary research study; this will be reported separately.

## 3 Results

The sample included 52 patients (35 women, 17 men) who had consulted with 34 different doctors (15 women, 19 men) across 12 GP surgeries in rural, urban, and inner-city areas in North London, the South West of England and the East of England. All interviews were conducted in English, and lasted between 26 and 97 minutes (average 58 min). Participants were aged between 19 and 96 years. 22 participants (42%) were over 64 years of age, 30 participants (58%) were aged between 19 and 64. Of the 52 participants, 45 identified themselves as White British (86.5%), three as White other, three as Black, and one as Asian. On average, 94.5% of communication items (box 1) were scored by patients as “good” or “very good”. Indices of deprivation of the participating practices were largely representative of practices nationally.

The fragments presented occurred during different moments of the interview, but commonly were prompted during discussions surrounding patients' reasons for consulting. The comments either specifically referred to the recent consultation which was being replayed on video, or related to past experiences with doctors. The content of consultations varied widely and represents the broad nature of general practice, from urgent appointments for new physical and mental health problems, to routine and follow-up appointments for long-term and life-limiting conditions.

We outline three threads common to the issue of ‘wasting doctors' time’ present across patients' narratives in general practice: 1) the experience of a conveyor belt approach to care, 2) the overt claim that ‘other patients’ waste time; beneath which lies 3) a prevailing uncertainty among patients over what is worthy of doctors' time.

### 3.1 Conveyor belt of patients

Patients spoke often of the pressured context in which healthcare encounters take place: the demand on services, the lack of time, and the busy doctors. This pressure was rendered explicit through personal experiences of being rushed through consultations:

“I mean you do sometimes get the feeling that they just want to get you out” (Nina, 65–74).

“I've been in and out faster than anybody would believe. They're obviously tired, or they're not interested and they just want to get rid of you” (Richard, 55–64).

“He makes you feel as if you're wasting his time. Before you've even sat down, y'know what I mean?” (Jackie, 55–64).

Esther had been invited in for a diabetes check-up. She saw the same doctor every time about her diabetes, as advised by the practice. She commented on the doctor's behaviour:

“I mean, he especially, makes me feel like a nobody. […] He doesn't say anything.[…] It's just his general attitude, you know, makes me feel like I'm wasting his time, not worth it” (Esther, 55–64).

A lady consulting with a long history of back problems explained how the doctor made her feel insignificant in the context of the long list of patients waiting to be seen:

“There's no body language there at all, no like concerns or anything like that, she's just like ‘oh god another one’. That's the expression I was getting from her. […] even though I was just trying to unload some of what was going on with me, she just didn't want to know. She's just like ‘oh next patient, next one’, conveyor belt going” (Kate, 35–44).

Patients experienced disappointment at the sense of being treated ‘like a number’ rather than a patient. One lady born abroad, consulted about a skin lesion and about symptoms relating to her rheumatoid arthritis. She recounted how, in her country of origin, there was more opportunity to develop rapport with the doctor, because the consultation ran at a more relaxed pace, unlike in the UK:

“It's almost like, oh yeah, another patient, what's wrong, what are you here for? […] But maybe they have so many patients that they're unable to do that” (Sandra, 65–74).

Beyond the time pressures on individual doctors, which interfered with consultation dynamics, burden on the healthcare system more widely was spoken of, and was presented as influencing decisions about consulting:

“There's a weird thing in the NHS where you're grateful for the service. So in that sense, you feel like responsible if you waste it” (Martha, 25–34).

“I also have a bit of an awareness of how stretched the NHS is and how you've kind of got to be, to a certain extent, grateful that it’s there […] This is a service with limited resources and, you know, effectively it's free and, you know, I think within that […] it's pretty good. But, you know, if you had all the time and money in the world it would be different” (Charlotte, 25–34).

These restrictions on resources influenced the relationship between patients and the health service, and fostered the feeling of timewasting. Martha saw the doctor who diagnosed a burst ear drum. She described the external pressures complicating her decision to see the doctor:

“There're these things kind of reminding people about why they don't need to go to the doctor, so then I think I want to feel like I'm actually going to the doctor for a reason. [...] Because often the reason why you're going to the doctor is because you know there's something wrong but you don't know what it is, but the kind of poster campaign things and the way the receptionist sometimes treats you, and the doctor, they kind of expect you to have a level of understanding about what you have and how it's treated before you go. [... ]So yeah, I suppose, it's a combination of maybe me just wanting not to waste their time, and being told that people that go to the doctors are constantly wasting their time. [...] Because I guess they must get a lot of timewasters if they're putting out all these posters. So you're trying to work out if you're a timewaster or not”

### 3.2 Using healthcare: the rational me, the irrational other

This reality of being rushed, alongside the explicit public message of pressure on health services, imposed the question of whether one was consulting ‘reasonably’ or not. In discussing their decisions to see the doctor, patients voiced their careful use of appointments. “I only go when I really need to” was a recurrent unprompted remark in the course of the interviews.

Janet was 96 and remembered when the NHS was introduced. She had known her GP for decades, and was very fond of him. She had recovered from cancer and had various other conditions for which she took several tablets every day. On this occasion, she was consulting about her medication and about some leg swelling. She enjoyed her appointments with her doctor because they often shared jokes together. However, she was keen to remind me that:

“Until I go back to see him when I've got to go for medical reasons and not a chit chat, I don't. I never go to him just for a chit chat [...] I only go when I need to go […] I never bother him with anything silly”

Similarly, Julie rarely attended her surgery. She visited her doctor about a recurrent ear infection and was referred to a specialist. She commented upon watching her consultation on video:

“I don't like going to the GP at all [...] and I don't go very often. I only go when I have to. And she obviously made me feel quite relaxed, ‘cos I can tell I look quite relaxed there” (age 45–54).

A mother with diabetes described how she only visited for ‘serious stuff’. Otherwise they were not a “doctory” family:

“Everybody says ‘Oh, go to the doctor's’ and that, but as I say, we don't. If we can sort things, we'll sort them ourselves. […] so we don't do trivial stuff and even with my children, I've never been taking them every five minutes” (Maureen, 55–64).

Several patients suggested that their upbringing had influenced their consulting behaviour and taught them not to rely on the healthcare system. These three women of different ages describe themselves as ‘rational users’ of services, displaying their compliance with tacit rules of good patient behaviour (Jeffery, 1979). Their own ‘good patient’ role was often defined in contrast to other patients who were seen to be less careful about their use of healthcare:

As a new mother attending the surgery more often with her toddler, Martha referred to other parents' behaviours:

“I think it's because I've had to go more, [laughingly] so I want to check that I'm not just being like a weird, like, over-reacting parent; which I'm sure they get a lot of”

An elderly lady, who was explicit in her cautious use, commented:

“I suppose some people go and waste doctor's time with things that are irrelevant” (relative of Joe, 75–84).

One man who had seen the doctor about some abdominal pain and leg pain, spoke of the new telephone triage system in the surgery, pointing out some of its benefits:

“Well it gets rid of the timewasters” (Jack, 75–84).

We see how the patient conjures up an image of self as responsible user, in contrast to other patients who are portrayed as ‘misusers’. This binary classification of rational and irrational user assumes a clear distinction between illness and health; those who need healthcare, and those who do not. It also assumes agreement between doctor and patient on what constitutes health and illness.

Public campaigns reinforce the good, rational healthcare-user profile by offering simple instructions to guide patients in their consultation choices. One such example in the UK is the NHS Choose Well campaign (Choose well this winter, 2013), which was designed to promote self-care and reduce burden of minor illness on general practice and accident and emergency. The posters list a series of common symptoms (back pain, stomach pain, chest pain, cuts and sprains), outlining a short management plan, indicating if and which healthcare service should be used. Whilst on the surface the instructions offered are straightforward, the terms used illustrate the complexity of what is asked of patients. The term ‘choose’, rich in terms of political currency, captures the freedom that patients hold over whether and when to seek medical opinion. However, the use of the imperative mode, combined with the adjective ‘well’, mitigates the notion of choice by urging the patient to behave in a way that is favourable to the healthcare system. Therein lies the paradox: choice is offered, but only to the extent that should the patient choose badly, moral labelling by healthcare providers may ensue. If the patient wishes to maintain a profile of ‘rational user’, choice becomes illusory.

### 3.3 Are my reasons good enough?

We have seen how patients present an approach to help-seeking which assumes an unproblematic transition from the healthy to the sick role (Heritage and Maynard, 2006). Public campaigns reinforce this outlook, although it is clear that choice to deviate may engender moral labelling, on the part of the doctor, other patients, and society at large. But what then of instances when doubt is cast upon the reasons to consult? What about when doctors and patients do not agree on the need for medical review? Or when demand on services is such that access becomes compromised? The neat dualism between rational and irrational user of healthcare lacks flexibility in its design when uncertainty arises over whether or not the patient's symptoms are deemed worthy of medical time.

Martha explained how the decision to see the doctor ‘only if absolutely necessary’ was not altogether easy. Her practice ran an access system as she termed ‘with no middle ground’. She could either book an emergency slot on the day, or she could book an appointment two weeks in advance. The process of ‘second guessing the diagnosis’ in order to then inform negotiations with reception staff was a difficult process. She referred to an earlier clinical encounter she organised for her son:

“Because you have to kind of self-assess whether you're an emergency or not I've started going to the pharmacy for advice […] So I did that with my son as well, [...] I didn't know if it was impetigo or not and I thought the pharmacy would have a better idea; so I said, oh, is there a cream that can treat him, and she said no, you need to take him to the doctor […]That probably sounds really weird, [...] I suppose it's my way of, like, reassuring myself that I'm not wasting the doctor's time or, you know, that it can't be resolved another way”

Here, seeking the input from another professional to assist in the decision-making process is central in validating the need for access. The pharmacist provides a source of authority and empowers the patient to request an urgent appointment.

Jack, a man in his late seventies with several chronic conditions, also thought it might be expected that he seek advice and obtain treatment from the pharmacist first. Indeed he was not one to go to the surgery unless “it was something really worthwhile”. On this occasion however, he chose to see the doctor:

“I thought, I've got to get it from the doctor, because if you start treating yourself, it becomes a problem. So that was one of the main things. But I felt that I might have been making a fuss. […] Yet it may be just indigestion, you know, here's me making a fuss and all the rest of it, and I've got indigestion. Although as I say, I felt pretty rough”

We see here the judicious weighing-up of what Jack perceives as trivial symptoms (“it is *just* indigestion”), for which he feels consulting would amount to fuss-making. And yet it is the associated physical malaise (“I felt pretty rough”) which finally prompts a visit.

Charlotte, who had pointed out that her family was not one to see the doctor, presented with abdominal pain and was referred for a scan. She explained her uncertainty over whether her reasons to consult were ‘good enough’:

“Yeah, I think it's just, because actually, especially for what I went, it's not like I'm, it's just a discomfort, it's not crippling pain or anything, [...] I obviously lead my life perfectly normally, so [...] do they just wonder if I'm being a hypochondriac. […] I suppose it's like going back to that time wasting thing as well, where you're not even sure if you should be there in the first place” (25–34).

She evokes this apprehension that the patient experiences in deciding whether the symptoms are worthy of doctors' time. The absence of “crippling pain” renders the presenting complaint more subject to scrutiny by the doctor. But having committed to walking into the doctor's consultation room, justifying her attendance becomes all the more crucial. This is complicated by what patients spoke of as the struggle to relay an accurate and relevant description of symptoms to the doctor. Marc was seeing the doctor about some symptoms which he suspected were caused by one of the tablets he was taking:

“I find it quite difficult to explain myself anyway, especially if there's something wrong with me” (Marc, 45–54).

Martha, Jack and Charlotte all provide accounts which contrast with the neat dualism of rational self and irrational other observed in earlier accounts. No patients felt they were, in the end, presenting to the doctor with trivial symptoms, but many disclosed the challenges in deciding whether to consult in the first place. This concern over consulting appropriately was also manifest among patients with several lifelong conditions. This reasonableness to consult is given consideration by the NHS Choices website (When to see your GP, 2014), an authoritative source for guiding patients through the national healthcare system in the UK. In assisting patients to make the best use of their doctor, the role of the GP is defined: “In some ways, the family doctor is like a social worker as they often deal with non-medical issues, such as housing, relationships or finances, which may be making you ill. GPs insist that their door is always open to any kind of problem. But are we making the best use of their time, and more importantly our own?” The commentary moves on to say those minor illnesses for which patients see the GP cost the NHS over £2 billion per year, hastily adding that patients presenting to the doctor should not be made to feel like timewasters or hypochondriacs. The GP interviewed reiterates: “if you just need a bit of reassuring, that's perfectly reasonable, this is our livelihood, it's what we do”. This passage further exemplifies the problems in defining a ‘good enough’ reason to consult, not only by patients, but by health providers as well. The message to patients to make suitable use of expensive resources is clear. At the same time, seeking reassurance is presented as a good enough reason to consult.

## 4 Discussion

In conversation with patients about their experience of general practice, we show that stories are constructed around a moral dualism of the rational and irrational user of healthcare. Explicit pressures on services recounted by patients frame these stories of cautious healthcare use. On the surface, public discourses reinforce the moral categorisation of consulting practices. However, this hides a more complex narrative of doubt, both among patients and in public campaigns, over what exactly constitutes rational consulting.

The ongoing struggle of general practice to define its professional remit complicates the question of appropriate help-seeking further.

Situated at the interface between the community and the healthcare system, the GP practice operates a policy wherein any reason deemed important enough to trigger a consultation by the patient is in principle endorsed as ‘good enough’. Despite this, a boundary is drawn, constituted by workload, money and time. The case of minor illness is particularly useful in understanding this boundary, because this debate has widespread repercussions on defining the chief responsibilities of general practice. Minor illness might traditionally denote what the doctors consider not to require input from the doctor, and what the patients worry their own symptoms will be branded as by the doctor should they decide to consult. In their study of time in general practice, Horobin and McIntosh describe the ambivalence doctors express when discussing their role in minor illness management (Horobin and McIntosh, 1983). Minor illness is presented, on the one hand as “wasted skills” and “intense boredom”, on the other hand, as a welcome break from the sometimes complex and emotionally-draining duties of general practice. This concern over the management of minor illness has a long history. Armstrong reminds us that the concern with “trivia” was already in existence in the early days of the NHS, and besides, was a central component in defining the core task of general practice (Armstrong, 1979). The GP was viewed as the doctor who deals with triage and trivia, whose role was presented in contrast to the hospital doctor for whom the exciting privilege of investigation, diagnosis and treatment was reserved. It was not until the late sixties that the GP enjoyed a renewed identity as the practitioner of biographical medicine, promoted to the status of attending to the person beyond the pathology (Armstrong, 1979). It is within this holistic definition of general practice that minor illness sits more comfortably, in particular if we consider that symptoms deemed barely worthy of medical attention on the surface may in fact be concealing more serious preoccupations or complaints. The formulation of the symptom iceberg (Hannay, 1980) provided some reassurance to GPs that a majority of minor ailments never presented to the doctor. And so the extent to which minor illness falls under the GP remit remains contested. The NHS choices website echoes this uncertainty and obscures the boundaries of rational consulting. With such ambiguity – for both doctors and patients – over what precisely lies within the realm of general practice work, the concern over wasting the doctor's time arises.

With the belief that other patients consult unnecessarily, there is a desire by patients to escape the moral label of ‘timewaster’, without necessarily knowing which presentations would be labelled as such by the doctor. With time acquiring the status of treasured commodity (MacBride-Stewart, 2013), the patient's decision to consult is put forward before the judgment of the doctor. Hillman's ethnographic study of a British emergency department describes how a moral judgment is assigned to patients as they are triaged according to perceived medical need (Hillman, 2014). In a context where “legitimacy is never assumed”, Hillman shows how patients must work to demonstrate their entitlement to the label of deserving patient. In general practice, Jones et al. describe the implicit categorisation of patients by general practitioners (Jones et al., 2004). May et al. highlight the process by which doctors form an evaluation of the patient in a way that potentially compromises the legitimacy of help-seeking (May et al., 2004). Our findings substantiate this view. We present patient perspectives which demonstrate that patients perceive the moral evaluation exerted by doctors in consultations. In turn, patients present themselves as idealised help-seekers, in contrast to ‘other’ timewasters. However, this obscures a deeper sense of uncertainty over what really constitutes appropriate help-seeking. Fischer and Ereaut go further: they describe fear as a central component of the doctor patient dynamic (Fischer and Ereault, 2012). One component of this fear they define as “entitlement anxiety”, namely an anxiety which is experienced by patients in anticipating an attendance where the doctor announces that the patient is not *really* ill, and that the patient did not *really* need to visit. Besides, any dichotomy between the good considerate user and the deviant, impulsive frequent user, assumes the existence of a reference ‘ideal’ user, which we have seen is elusive to doctors and patients. Bloor and Horobin defined the *double-bind* predicament several decades ago, writing: “It would seem then that doctors tend to typify the ideal patient as someone who is able to assess symptomatology with sufficient expertise to know which conditions he should present, and when he should present them to the GP, but at the same time one who, having assessed his condition, will defer to the doctor's assessment on presentation” (Bloor and Horobin, 1975, p. 276). Although the paternalistic model of the doctor-patient encounter is outmoded, the act of deferring to an expert imposes a power differential which inevitably leaves the patient in a position of vulnerability. The persistence of the adjudication act that more or less subtly accompanies any consultation may serve to intensify the power differential. Whilst some degree of power asymmetry may be inherent to the encounter and is not necessarily problematic (Pilnick and Dingwall, 2011), the process of casting a moral evaluation adds the potential for ‘dysfunctional’ asymmetry. Casting a moral evaluation of ‘timewaster’ amounts to an act of stereotyping. Coyle describes the disempowering “personal identity threat” that patients experience as a result of the routine stereotyping by doctors (Coyle, 1999).

## 5 Candidacy

We have seen how an understanding of healthcare to be in short supply, arising from GP consultation experiences and public discourses, acquires dominance in patients' narratives as a moral concern about timewasting. Contradictions inherent to the GP role may confuse the social discourse further, and are reflected in patients' accounts of negotiating service use.

**Fig. 1:**
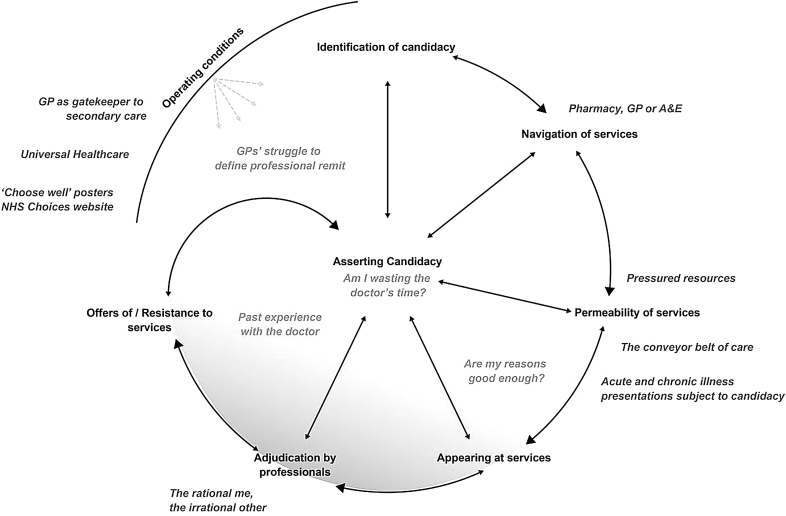
**- Asserting candidacy in primary care.** The stages of candidacy are represented in black. Asserting candidacy typically follows a staged sequence from ‘identification’ to ‘offers of/resistance to services’. It is a dynamic model with each item interlinked and influencing others, represented by the small arrows. All stages contribute to ‘asserting candidacy’. The ‘operating conditions’ bear upon all stages of the model. The inner circle shadowed area represents the covert dimensions of asserting candidacy; the more overt factors contributing to asserting candidacy are depicted in dark grey.

Studies of help-seeking in cancer have repeatedly identified the presence of ‘fear of wasting doctors’ time’ as a possible factor contributing to delay in visiting the doctor (Corner et al., 2006, Forbes et al., 2014). In psychology, models of help-seeking have been developed focussing on cognitive and emotional factors which inform consulting behaviours (Wyke et al., 2013). Sociological models have moved beyond individual factors to consider social and structural health system processes which alter consulting practices (Wyke et al., 2013). However, the moral question of cautious healthcare use which we encounter in our interviews is absent from these models. Dixon-Woods and colleagues' notion of candidacy, which is born out of a critical interpretive review of healthcare access among vulnerable groups, offers a helpful framework here (Dixon-Woods et al., 2006). Candidacy is a model which maps out the patient's journey as six stages through healthcare: 1) identification of candidacy, 2) navigation, 3) permeability of services, 4) appearances at health services, 5) adjudication by professionals, 6) offers of/resistance to services. Candidacy is a move beyond traditional measures of access, to understand the staged process by which the patient becomes a candidate for seeking healthcare, and negotiates legitimacy as a patient when entering into dialogue with the healthcare system. In doing so, candidacy succeeds in displaying more vividly the moral dimension of help-seeking. Each stage contributes toward asserting candidacy. The concept makes allowance for the complexity and interdependence integral to the process of consulting, without inevitably referring to a comparison group of ‘good’ users. The model sheds light on the covert state of uncertainly expressed by participants in our interviews over what problems might be worthy of doctor's time or not (‘are my reasons good enough? ‘Am I wasting the doctor's time?’); an uncertainty hitherto obscured by a dominant talk of good and bad users of healthcare (‘the rational me, the irrational other’). Fig. 1 illustrates the stages of candidacy and displays the relevance of the model to our findings. ‘Navigating services’ in our stories is illustrated by participants approaching pharmacists for advice on best routes to care. The ‘Choose Well’ Campaign also seeks to facilitate this process. ‘Permeability of services’ refers to the degree of difficulty required to access a service. General practice is in principle a highly ‘permeable’ service given its premise of a free ‘open door’ policy. However, the reality of demand on services, as we have seen through the accounts of our participants, can render an appointment with the doctor hard to come by.

The stages of ‘appearances at health services’ and ‘adjudication’ are particularly relevant to our argument, because they encompass the notion of ‘asserting entitlement’ which is important in our narratives of patients' help-seeking. Dixon-Woods et al. consider how ‘appearance at health services’ requires the skills to assert one's claim to candidacy, which entails the ability to voice one's credibility (Dixon-Woods et al., 2006). Having initiated contact with the health service, the next step is ‘adjudication’ – the “judgments and decisions made by a professional which allow or inhibit continued progression of candidacy”. The authors argue that ‘appearing at health services’ may sit more comfortably with the middle classes. Whilst legitimacy is indeed likely to be more laboriously acquired by the socially deprived, our interviews suggest that the search for legitimacy is prevalent more widely, especially in circumstances where demand for care exceeds supply. The staged process of candidacy thus becomes relevant to most primary care encounters, albeit in a more subtle form, because each stage is not necessarily explicitly contested. We suggest candidacy therefore applies beyond vulnerable populations to all patients in primary care who acquire vulnerability merely by enacting the patient role. Notably, those patients presenting with isolated symptoms, as well as those discussing issues relating to longstanding chronic disease, spoke of their worry about wasting the doctor's time. Many spoke of their struggle to articulate the relevance of their complaints to the doctor. Whilst it may seem surprising that the chronically ill patients, whose attendance is expected, still experience concerns about appropriate help-seeking, Strong observed similar concerns among parents attending a hospital paediatric clinic. One would anticipate that the prerequisite referral from primary care would erase any adjudication of help-seeking at secondary care level, and yet Strong refers to parents' struggle to know what “would count as a proper medical problem worthy of staff's consideration” (Strong, 1979b, p. 159).

By recognising the struggles involved in asserting candidacy in the face of looming adjudication, Dixon-Woods et al. provide a framework that elucidates clearly how moral economies of entitlement contribute to help-seeking. Our participants' accounts illustrate how dynamics within the consultation, explicit pressures in surgeries, public discourses urging cautious use, all contribute to characterising candidacy in primary care. Candidacy acknowledges relevance at the micro doctor-patient relationship level, whilst also considering ‘operating conditions’, namely the wider social and cultural context of the encounter, including allocation of resources and configuration of services. We refer to a UK context which is one of primary care underfunding (Roland and Everington, 2016), increasing workload (Hobbs et al., 2016), and low levels of professional satisfaction (Gibson et al., 2015), which in turn intensify the patient's need to assert candidacy. Studies in US primary care also report the patient's preoccupation to establish the reasonableness of consulting (Heritage and Robinson, 2006). The phenomenon of worry about timewasting, although not a uniquely British phenomenon (Forbes et al., 2013), may be more pronounced in a country which pledges to provide free universal healthcare. As such, a unit of care acquires value, in so far as spending allocated to one unit of care results in loss elsewhere. It may be that the operating conditions of countries where a financial transaction takes place between patient and doctor, outside the constraints of a defined budget, may not engender such a cautious appraisal of welfare entitlement, thus compromising the degree to which candidacy requires assertion. The gatekeeping role of the GP in the UK means that an unselected range of conditions present to the GP compared to countries with no gatekeeping, perhaps lending itself to a higher possibility of doctors adjudicating on merit of presentation.

Candidacy offers a more dynamic definition of appropriate health service utilisation, one that is negotiated and agreed between doctor and patient. Whilst the health service and the patient are in parallel seeking to establish “the appropriate objects of medical attention and intervention” (Dixon-Woods et al., 2006), candidacy describes how the eligibility of the encounter becomes defined by both parties.

## 6 Methodological considerations

### 6.1 Limitations

The subject area was not a primary aim of the original investigation. It was therefore not explicitly prompted for during interview, and so when it did not emerge, we cannot say whether its absence amounts to the subject not being of significance to these participants, or whether the conversation simply took a different direction. Whilst the presence in patients' narratives of this concern over timewasting was prominent enough to merit independent study, the nature of the analysis limits any aspiration to achieve saturation (O'Reilly and Parker, 2013). We did not formally document the type of appointments. Presenting complaints encompassed acute and chronic conditions and were broadly representative of a British general practice population. The retrospective categorisation by the researcher of consultations is fraught with challenges (Salisbury et al., 2013), and complicated by the several and frequently overlapping problems discussed, as is typical of general practice consultations. Whilst some patients attended following an invite from the practice, these consultations regularly dealt with more than one issue, so the question over whether to raise symptoms or not with the doctor remains relevant. Our method of data collection excluded doctors and patients who declined to be videoed. Of those who consented to be videoed, a minority agreed to be contacted for interview. Participation in an interview may be a daunting prospect. Indeed the readiness to share personal outlooks and experiences with a stranger is more likely to appeal to certain temperaments. The addition of the video complicates the interview process. Although valuable in providing depth and specificity, the video may further alienate some otherwise interested participants.

Our design is such that we are not able to present the doctors' views on the patient's consulting practices. We focus here on providing the patients' perspective. We cannot comment on whether doctors were indeed adjudicating in these particular instances. We rely on published studies of doctors' views to corroborate our findings. While this provides a one-sided view, it is largely patients' beliefs and experiences which guide entry into the health service. Furthermore, the accounts of patients we present offer insight into doctors' past and present behaviours.

For purposes relating to the primary aims of the study, our cohort of practices ranked in the lowest quartile nationally on communication in the GP patient survey (Roland et al., In Press). However, lower scoring practices include doctors who individually score well on communication (Roberts et al., 2014). It is likely that, within our cohort of poor scoring practices, doctors who were more confident about their communication skills were more willing to agree to video-recording of their consultations. The survey results in our study support this hypothesis. Patients on average scored 94% of communication items as good or very good. These results are in line with national survey averages. This suggests that the consultations in this study are likely to be typical of general practice consultations more widely.

### 6.2 Strengths

The analytical approach offers strengths. The spontaneous emergence of data cannot – by design – be explicitly solicited by the researcher, and thus it can be argued that the findings are less prone to social desirability bias. It is likely that the added visual stimulation offered by the observation of the patient's consultation during the interview triggered the discovery of supplementary matters for discussion. The use of the video-elicitation interview generated multi-layered patient accounts of healthcare experience, offering generous data yield. In line with grounded theory principles, themes arising from the data which are not pre-imposed on participants are particularly worthy of attention, especially when they are recurrent across several interviews. Its absence from the topic guide means the issue of wasting time is all the more likely to be a *true* concern among the participants who spoke of it, and its natural occurrence makes it less likely to be a product of the interview artefact. The phenomenon of serendipity is an established and encouraged process in qualitative research. In writing on the value of serendipity in ethnographic research, Rivoal and Salazar quote the sociologist Merton: “[serendipity] involves the unanticipated, anomalous and strategic datum which exerts pressure upon the investigator for a new direction of inquiry which extends theory” (Rivoal and Salazar, 2013). That said, no conversation is exempt from the ordinary obligations inherent to any social transaction. There may be some element of social pressure to appear – even in the interview context – as a ‘reasonable service user’. It is accepted that any interview data arise as a co-creation of the encounter between researcher and participant, a process which does not necessarily negate the findings presented. Moreover, the literature confirms that the issues discussed here are relevant to patients beyond the sample interviewed.

### 6.3 Reflexivity

In line with principles of reflexivity, it is important to acknowledge the authors' background. As a practising GP, NL may have been more attuned to detecting and collecting accounts of concerns over timewasting in the dataset. In striving for objectivity as far as possible, she took care to avoid divulging her clinical identity during interviews, to avoid potential influence upon participants' responses. She worked in collaboration with social scientists (JN), health services researchers (JB), and clinicians (JC, MR) throughout data collection, data analysis and preparation of this manuscript to ensure an accurate and balanced interpretation of findings.

## 7 Conclusions

Whilst a proportion of patients with chronic conditions will receive invitations to make contact with their doctor, patients in general practice still largely hold the responsibility for initiating the encounter and disclosing their symptoms to the doctor. Our interviews have provided some insight into how patients enact this process, and the challenges they meet in doing so. We have seen how patients experience added moral pressure to ‘choose well’ in a cultural context in which healthcare is conceived as a limited good in short supply. In the midst of these choices, the worry arises about wasting the doctor's time, where time is conceptualised as a limited resource which needs considered allocation. There is a long history in the UK of doctors feeling patients should be more discerning in their decisions to consult. The historical relevance of trivia in the crisis of identity that general practitioners experienced earlier on in the twentieth century, reminds us that the role of the community doctor shifts back and forth between one resembling that of social worker to that of investigator of organic disease. Contradictory social discourses echo this ambiguity. While the message on the surface seems clear – to consult at the *right time*: not too early to be inappropriate and waste time, but not too late to be seen to be neglecting one's health needs – we have examined the struggles involved in enacting this request, given that appropriate need is so difficult to define. We suggest the concept of candidacy as one approach which moves the discourse beyond the dichotomy of the responsible and irresponsible user.

Worry about timewasting is a phenomenon that deserves attention for several reasons. First, at the micro-level of the consultation, being attuned to the possible presence of these dilemmas patients grapple with could help foster a more subtle understanding of the patient's experience of illness and, accordingly, improve the quality of the encounter for both patient and doctor. Second, at the macro-level, an awareness of this phenomenon could refine how the health service communicates with patients, and empower patients to seek help rather than worry about timewasting, ultimately leading to an improved experience of healthcare for patients, and, in some instances, a more timely diagnosis.

## Box 1 GP-patient communication items

Alt-text: Box 1

## Box 2 The video-elicitation interview

Following introductions, explanations and consent, the interviewer asks the participant background questions about the doctor and the consultation: Have you seen this doctor before? Have you seen a doctor about this problem before? Is this an ongoing problem? Has the problem settled now? The videoed consultation is then played on the researcher’s laptop, and the patient is encouraged to pause and comment on any aspects of the consultation, including how he/she was feeling at the time, how the doctor behaved, what the doctor said. The viewing of the videoed consultation is used as a means to elicit reflection and aid explanation by the patient for his/her responses to each item on the communication questionnaire. The interview follows the order of the questions on the questionnaire (box 1). A theoretical example: “I gave the doctor *very good* for ‘giving you enough time’ because he made me feel very comfortable by turning toward me, and avoiding looking at the computer screen. He didn't interrupt me, and he was very sympathetic in his demeanour.”

Alt-text: Box 2
